# Photobiomodulation Elicits a Differential Cytokine Response in a Cultured Analogue of Human Skin

**Published:** 2019-03-01

**Authors:** Nicholas J. Prindeze, Jeremy G. Ardanuy, Bonnie C. Carney, Lauren T. Moffatt, Jeffrey W. Shupp

**Affiliations:** ^a^Firefighters’ Burn and Surgical Research Laboratory, MedStar Health Research Institute, Washington, DC; ^b^The Burn Center, Department of Surgery, MedStar Washington Hospital Center, Washington, DC

**Keywords:** photobiomodulation, light therapy, 3-dimensional culture, skin substitute, wound healing

## Abstract

**Background:** The study of photobiomodulation in wound healing is encumbered by limited wound study models. The aim of this study was to investigate the efficacy of a 3-dimensional dermal tissue culture model as a cost-saving alternative to conventional photobiomodulation study techniques. **Methods:** Nine dermal analogue tissue cultures were treated for 2 days with sham or 660-nm wavelength of light at either 1.5 or 3 mW/cm^2^ of energy. Tissue cytokine mRNA production was assessed by real-time reverse transcription-polymerase chain reaction, and tissue and supernatant protein were evaluated by immunofluorescence, enzyme-linked immunosorbent assay, and Western blot. **Results:** Photobiomodulation with 660-nm wavelength light induced transcription of IL-1β and IL-6 mRNA and decreased that of IL-8. Tissue protein content of IL-6 and IL-8 was unchanged, whereas supernatant protein content of IL-8 was significantly increased (*P* = .023) by 1.5 mW/cm^2^ treatment. To describe the localization of cytokines between tissue and supernatant, the relative diffusion of each was calculated and found to be 15-fold higher for IL-6 than for IL-8 despite an overall higher concentration of IL-8 in the tissue. **Conclusion:** In this study, photobiomodulation elicited mRNA and protein changes quantifiable in both the tissue and supernatant. In addition, the use of this advanced culture model allowed for histological assessment and the comparison of “local” versus “circulatory” responses between the tissue and supernatant, respectively.

Following the development of the laser in the 1960s and the high-power LED in the 1970s, the applications of light therapy have grown expeditiously to span the fields of chronic pain, inflammation, and nerve and tissue regeneration.^1^^-^3 Wound healing has emerged as a major field to harness the advantages of light,^3^ especially in the therapies of burn injuries,^4^ diabetic and chronic wounds,^5^ scars,^6^ and a host of other lesions.^3^^,^^7^

While the number of light application studies in wound healing has increased from 90 in the year 2000 to more than 200 in 2013, a fundamental drawback still exists: wounds are complicated systems, and wound healing involves multiple phases and a host of local and systemic factors.^8^^,^^9^ For this reason, the study of intricate pathways or mechanisms becomes exponentially more difficult in an in vivo model system, detracting from the basic science. Conversely, single cell type or coculture in vitro models do not offer a complete biological representation of wound-healing processes due to the lack of differentiated tissue structure, multicellular interactions, and systemic influence present in vivo.

Recent developments in in vitro tissue culture have resulted in 3-dimensional full-thickness analogues of human skin composed of dermal keratinocytes and fibroblasts. These analogues are lower in cost than animal models, present more sophisticated cellular diversity to better mimic that found in skin, and are capable of being injured and healed^10^^,^^11^ (Fig 1). These models have found use in a variety of fields including wound healing,^12^ cancer,^13^ and drug testing.^14^

Interestingly, these culture models conserve the anatomy of the keratinocyte and fibroblast structure of the epidermis and the dermis.^10^ They also maintain similar nutrient delivery mechanisms where nutrients and waste diffuse passively through the surrounding matrix and between the culture reservoir and the tissue, an analogue of dermal circulation.^10^ These models also contain a mature basement membrane, including hemidesmosomes with a well-developed lamina lucida and lamina densa, and tonofilaments extending into the cytoplasm.^15^ Therefore, this model is useful for the study of fully differentiated skin and the local and systemic effects of a stimulus as well.

It is our hypothesis that the use of a dermal analogue constitutes a more reliable and informative means to study photobiomodulation (PBM) over the simplistic cell culture models. Similarly, it offers a practical alternative to costly and complicated animal systems. In this study, the response of a cultured full-thickness human skin analogue to treatment with 660-nm wavelength light was examined. Treatment with 660-nm wavelength light was chosen for these experiments since it is commonly used in PBM wound and burn studies, such as time to wound healing, neovascularization, and growth factor expression, among others.^16^^-^19 The “local” and “systemic” responses of the analogues were assessed using real-time reverse transcription-polymerase chain reaction (RT-PCR), histological and Western blot analyses for the tissue cytokines, and enzyme-linked immunosorbent assay (ELISA) and Western blot for cytokines in the supernatant.

## METHODS

### Study design

Nine full-thickness EpiDermFT (MatTek, Ashland, Mass) human skin analogues composed of keratinocytes and fibroblasts were grown in culture in a humidified human cell culture incubator with 5% CO_2_ supplement. Proprietary, serum-free culture media was provided by the manufacturer (MatTek). Treatment groups were defined as follows: 1.5 mW/cm^2^ treatment (n = 3), 3 mW/cm^2^ treatment (n = 3), and sham (n = 3). Sham cultures were handled identically to the treated cultures and placed on the treatment stage but were not treated with light. Following a 2-day incubation period, baseline media was collected and stored at −80°C. Analogue tissue was incubated for an additional 2 days with light treatment or sham on days 2 and 3, precisely 48 and 72 hours from culture initiation. On day 4, the media was collected, a biopsy was preserved in an optimal cutting temperature (OCT) embedding medium (Tissue-Tek, Torrance, Calif) for histology, and remaining cells were preserved in TRIzol (Life Technologies, Grand Island, NY) for nucleic acid and protein analyses.

### PBM treatment

PBM treatment was delivered to the epidermal side of the skin analogue tissue from an array of LEDs (NTE Electronics, Bloomfield, NJ). LED peak emission wavelength was 660 nm, with a spectral line half-width of 20 nm. LED power output was calibrated with the use of a ThorLabs (Newton, NJ) optical power meter and an associated 50 nW-50 mW, 200-1100 nm rated photodiode probe. Calibration was performed at the surface of the treatment stage, in the cell culture incubator, under normal temperature and humidity conditions, protected from room light. While not undergoing treatment, samples were stored in the same incubation chamber, separated and blocked from the treatment device by an opaque black foil barrier. Light was delivered at 1.5 mW/cm^2^ for 44.4 minutes or 1.5 mW/cm^2^ for 22.2 minutes for a conserved delivery of 4 J/cm^2^.

### RNA isolation and real-time polymerase chain reaction

Biopsy samples preserved in TRIzol (Life Technologies) were homogenized in a Qiagen TissueLyser LT (Venlo, the Netherlands). RNA was subsequently precipitated with chloroform overnight at −80°C per the manufacturer's protocol. Sample quantity was determined with a Nanodrop 2000c spectrophotometer (ThermoFisher, Waltham, Mass), and quality was assessed using the sample absorption ratios at 260 nm/280 nm.

Real-time RT-PCR and analysis were performed as previously described.^20^ Briefly, RNA samples were assayed using the iScript One-Step RT-PCR Kit with SYBR green (Bio-Rad Laboratories, Irvine, Calif) with oligonucleotide primers (Table 1) (Integrated DNA Technologies, Coralville, Iowa). Glyceraldehyde 3-phosphate dehydrogenase (GAPDH) was used as a reference gene, and levels were quantified in all samples in parallel with target genes. Reactions were run in a Real-Time PCR Detection System (Bio-Rad Laboratories). Normalization was calculated relative to the GAPDH housekeeping gene using the Δ*C*_T_ method.

### Protein isolation and quantification

Following the removal of RNA from the phenol-chloroform extraction, DNA was removed with ethanol and protein was precipitated with isopropanol overnight at −80°C. Precipitated total protein was washed 3 times with 0.3M guanidine hydrochloride in 95% ethanol for 20 minutes each, followed by 1 wash with 100% ethanol per the manufacturer's protocol for dual RNA and protein isolation. Protein pellets were then solubilized in 8M urea solution containing 1% protease inhibitor cocktail (Sigma, St Louis, Mo) at 55°C for 20 minutes. Urea was then gradually exchanged for a Tris-HCl buffer with 1% Triton-X through serial dilution and concentration in a 3-kDa Amicon ultra centrifuge filter (Millipore, Darmstadt, Germany).

The Coomassie Plus Bradford Assay (Thermo Scientific, Rockford, Ill) was used to quantify total protein following standard protocol. Briefly, a standard curve was prepared by dilution of bovine serum albumin (BSA) protein (Sigma, St Louis, Mo) in Tris-HCl buffer. Five microliters of standard or isolated total protein was mixed with 150 μL of Coomassie Plus reagent (Thermo Scientific) in a microtiter plate and absorbance was assessed at 570 nm.

### Immunoprecipitation

Forty nanograms of isolated total protein was incubated with 1 μg of mouse IgG1 anti-human IL-6 or IL-8 primary antibody (Abcam, Cambridge, England). Antibody-protein complexes were then incubated with 1.5 mg of protein G-bound magnetic beads (Life Technologies). Bead-antibody-antigen complexes were washed with PBS and protein was eluted with a glycine buffer at pH 2.8. Excess antibody and beads were used relative to starting protein to ensure a complete protein capture.

### Western blot

Protein samples were denatured with Laemmli buffer (Bio-Rad), run on a 4% to 20% TGX gel (Bio-Rad), and then transferred to a nitrocellulose membrane (Bio-Rad). Membranes were probed overnight with anti-IL-6 or IL-8 rabbit antibodies (Abcam) as a primary antibody, followed by goat-anti-rabbit antibodies conjugated to HRP as a secondary antibody. SuperSignal West Dura chemiluminescent substrate (Thermo Scientific) was used for development, and images were captured with a Gel Logic 2200 imager (Kodak, Rochester, NY).

### Enzyme-linked immunosorbent assay

Supernatant and isolated tissue samples were assayed for IL-6 and IL-8 content by SABioscience (Qiagen) ELISA kit following standard protocol. Briefly, samples and protein standards were bound by the sandwich capture method with plate-adhered primary antibody, biotin-conjugated primary antibody, and secondary HRP-conjugated avidin HRP. Catalysis of 3,3′,5,5′-tetramethylbenzidine (TMB) to TMB diamine was detected by absorbance at 450 nm in a Perkin Elmer Victor 3 (Waltham, Mass) plate reader with background subtraction at 570 nm.

### Immunofluorescence

OCT-embedded biopsy samples were cryosectioned and adhered to positively charged slides. Slides were fixed in 100% ethanol, blocked with 1% BSA and 5% milk, and stained with either mouse-anti IL-8 or rabbit-anti IL-6 primary antibodies (Abcam). Goat anti-mouse Cy3 or goat anti-rabbit Cy5 antibodies (Abcam) were used for secondary detection. Control sections were prepared by incubating tissue without primary antibody to confirm the absence of background staining. Images were captured with a fluorescent Zeiss Axio Imager microscope (Jena, Germany).

## RESULTS

### Tissue mRNA transcript analysis

**Figure 2:**
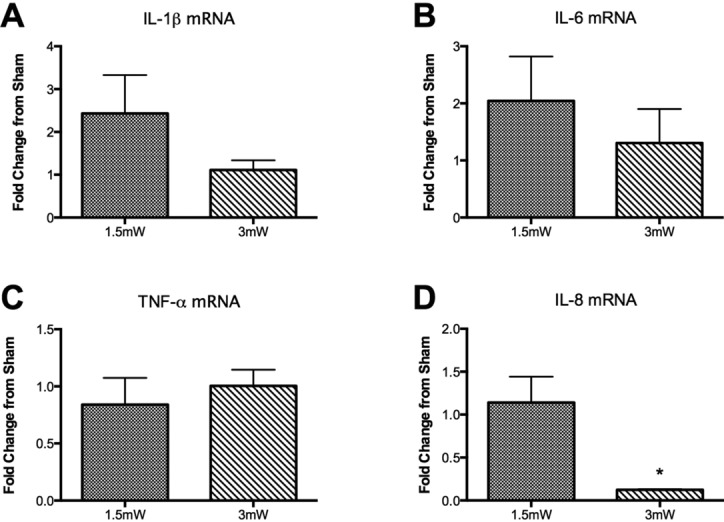
mRNA expression data for cytokines (*a*) IL-1β, (*b*) IL-6, (*c*) TNF-α, and (*d*) IL-8. Data expressed as mean ± SEM. Significance assessed by Student's *t* test. *P < .05 from sham.

Following 2 days of treatment with 660-nm wavelength light, tissues exhibited a dose-dependent response in mRNA transcription (Fig 2). Treatment with a power density of 1.5 mW/cm^2^ of light produced a minor upregulation of cytokines IL-1β and IL-6 expression compared with sham (Figs 2*a* and 2*b*), while the 3 mW/cm^2^ treatment resulted in no change. IL-8 transcription (Fig 2*d*) was unaffected by 1.5 mW/cm^2^ treatment but significantly decreased with 3 mW/cm^2^ treatment (*P* = .002). Both treatment fluencies produced no change in TNF-α mRNA expression (Fig 2*c*). All transcript data are normalized to the reference gene and expressed as a fold-change from sham treatment.

### Tissue protein analysis of select cytokines

**Figure 3:**
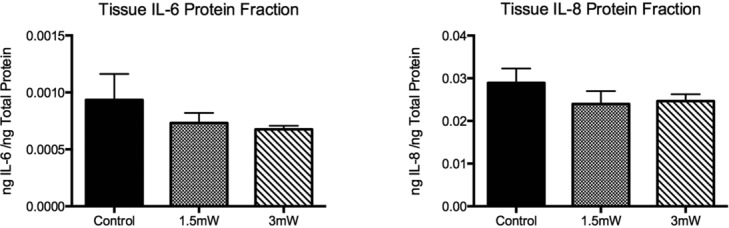
Tissue IL-6 and IL-8 content as assessed by ELISA, and normalized to total protein content as assessed by the Bradford assay. Data are expressed as mean ± SEM. Significance assessed by Student's *t* test.

**Figure 4:**
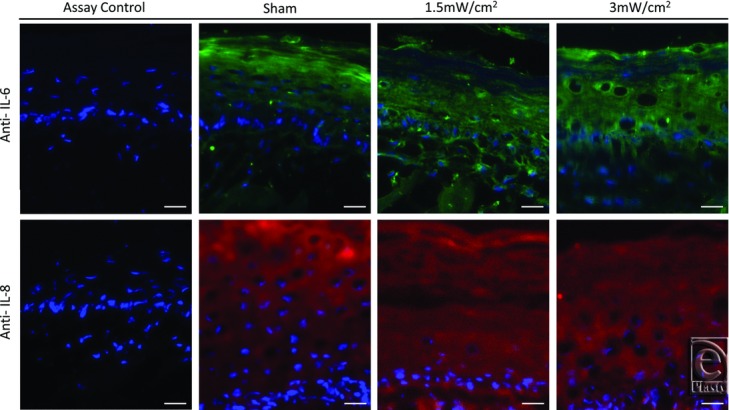
Immunofluorescent localization of IL-6 and IL-8 within analogue tissues. Control sections were stained in the absence of primary antibody. Scale bar represents 25 μm.

Isolated tissue protein, quantified by ELISA, was normalized to the total protein content of the tissue extracts (Fig 3). IL-6 and IL-8 were present in the tissue at approximately 0.3 and 10 ng/mL, respectively, by ELISA, demonstrating a 30-fold difference between IL-8 and IL-6 levels. Both IL-6 and IL-8 levels in the tissue were unchanged in treatment groups compared with sham. Histological and immunofluorescent analyses confirmed this result and illustrate a diffuse, low concentration of both cytokines, particularly throughout the epidermis (Fig 4).

### Supernatant protein analysis of select cytokines

**Figure 5:**
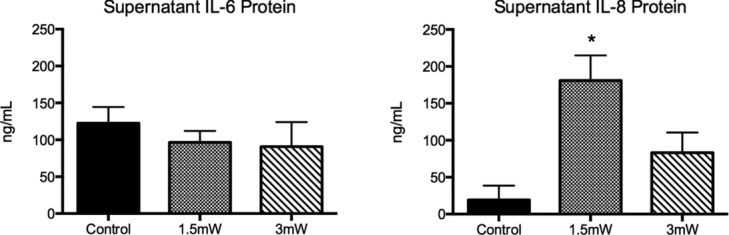
Supernatant IL-6 and IL-8 cytokine content as assessed by ELISA. Data are expressed as mean ± SEM. Significance assessed by Student's *t* test. *P < .05 from sham.

**Figure 6:**
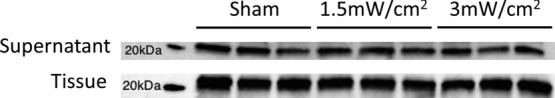
Representative Western blot of IL-6. Note that these blots are performed with 0.5 and 1.5 ng/mL of primary antibody for supernatant and tissue protein, respectively.

ELISA of tissue culture supernatant for IL-6 revealed similar protein content in the treated and sham groups (Fig 5), which was confirmed with Western blot (Fig 6). Conversely, the supernatant content of IL-8 increased in both treatment groups over sham, which was statistically significant (*P* = .023) for 1.5 mW/cm^2^ treatment. Assessment of supernatants showed no detectable IL-1 β in any treatment group or sham by ELISA. The lowest limit of detection of IL-1β in the assay was 17.9 pg/mL

### Diffusability of skin analogues to IL-6 and IL-8

To characterize the difference in diffusion from the tissue to the supernatant of each cytokine, a simple diffusability value was calculated by equation 1.


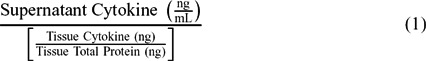


**Figure 7:**
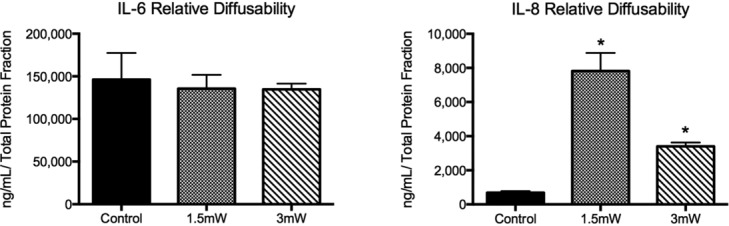
Supernatant and tissue IL-6 and IL-8 relative diffusability. Data generated by dividing supernatant content by tissue relative content for each cytokine. Data are expressed as mean ± SEM. Significance assessed by Student's *t* test. *P < .05 from sham.

The diffusability of IL-6 and IL-8 is approximately 150,000 and 10,000, respectively (Fig 7). These results highlight selective retention and/or diffusion of the tissue scaffold, as IL-6 diffused to produce a 15 times sharper gradient between the 2 culture phases in comparison with IL-8.

## DISCUSSION

Clinical interest and the number of studies in the field of PBM have increased considerably in recent years without advancement of available model systems. In this study, the effects of PBM on a new multidimensional culture model were examined in an effort to introduce lower cost and more targeted assay systems to this growing field. Results were consistent with previous PBM studies, as well as histological and regional comparisons not previously possible with traditional in vitro culture were identified. Light treatment parameters used for this study were modeled from standards commonly found in other reports.^21^^-^23 The results we obtained included transcriptional changes of cytokines IL-1β, IL-6, and IL-8, as well as an increase in IL-8 protein. Although the application of this model is novel, these results correlate with those of several conventional in vivo and in vitro studies.^21^^-^29

One of the major challenges of PBM studies is the accurate determination of treatment fluency or energy delivered per surface area. In many studies, this can be difficult to calculate or maintain due to variation in distance from a treatment surface or use of a nonhomogeneous source. However, with this model, PBM treatment was easily delivered to the epidermal surface in a controlled manner, with a consistent treatment fluency and without confounding factors such as hair interference, surface contamination, or patient movement that may impact in vivo or clinical studies.

Another important and useful aspect of this model is the compartmentalization of separate tissue and media phases. Since cellular products must diffuse through the tissue extracellular matrix to reach the media, including the epidermis, assessment of these phases may accurately simulate a local versus systemic circulation assessment. In the present study, IL-8 content in the tissue was present at a 30-fold higher concentration than IL-6 whereas the difference in diffusion to the media phase was approximately 15 times higher for IL-6. These differences can be explained by the chemokine function of IL-8 30^,^^31^ relative to the systemic signaling function of IL-6 32^,^^33^ and from their respective physical characteristics (Table 2). However, the ability to describe and quantify tissue protein diffusion and characterize modes of signaling may be a significant advancement in in vitro culture utility.

The most unique aspect of this tissue culture model may be the ability to biopsy and examine histologically tissue architecture and protein localization. Although it was discovered that paraffin embedding results in problematic tissue integrity, cryotomy produced high-quality sections, readily assayable by immunohistochemostry. In this study, IL-6 and IL-8 tissue content ELISA data could be confirmed by immunofluorescence, as well as the epithelial location determined and visualized. This feature of the model adds a significant degree of information to studies of protein movement, association, and cellular interaction.

## CONCLUSION

The results of this study highlight the utility of new, multidimensional culture models and support their use for the study of PBM and other fields of light therapy. These model systems are cost-effective, serve as an analogue of local and circulatory responses, and may be assayed by histology. Their use may allow for a further understanding of sensitive wound-healing pathways stimulated by PBM by eliminating the complication and oversimplification of in vivo and in vitro systems, respectively.

## Figures and Tables

**Figure 1:**
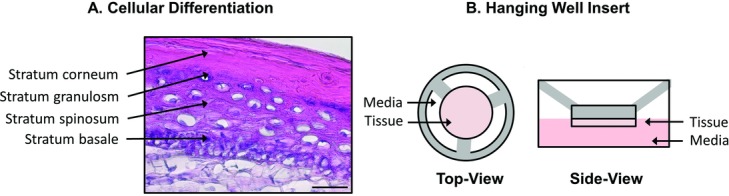
(*a*) Representative hematoxyline-eosin–stained photomicrograph illustrating epidermal differentiation. Scale bar represents 200 μm. (*b*) Illustration of the hanging well-insert tissue scaffold.

**Table 1:** Primer sequences of mRNA analytes

Gene	Primer sequence	Annealing temperature, °C
IL-1β	F-5′-AGA-TGA-TAA-GCC-CAC-TCT-ACA-G-3′	50
	R-5′-ACA-TTC-AGC-ACA-GGA-CTC-TC-3′	
IL-6	F-5′-ACA-GCC-ACT-CAC-CTC-TTC-AG-3′	45
	R-5′-CCA TCT TTT TCA GCC ATC TTT-3′	
IL-8	F-5′-ATG-ACT-TCC-AAG-CTG-GCC-GTG-GCT-3′	58
	R-5′-TCT-CAG-CCC-TCT-TCA-AAA-ACT-TCT-C-3′	
TNF-α	F-5′-CCC-GAG-TGA-CAA-GCC-TGT-AG-3′	50
	R-5′-GAT-GGC-AGA-GAG-GAG-GTT-GAC-3′	
GAPDH	F-5′-CAA-TGA-CCC-CTT-CAT-TGA-CCT-3′	50
	R-5′-AGC-ATC-GCC-CCA-CTT-GAT-T-3′	

**Table 2:** Physical characteristics of protein analytes

Parameter	IL-6	IL-8
Molecular weight, kDa	23-28*^32^	8^33^
Isoelectric point, pH	4-5.3^34^	8.65^35^
Tertiary structure	4 α-helices^36^	2 α-helices, 1 β-sheet^37^

*The molecular weight of IL-6 is known to vary on the basis of posttranslational modification.
